# Adipose-Derived Mesenchymal Stem Cells Reduce Lymphocytic Infiltration in a Rabbit Model of Induced Autoimmune Dacryoadenitis

**DOI:** 10.1167/iovs.15-17824

**Published:** 2018-06

**Authors:** Xue Li, Xiaoxiao Lu, Deming Sun, Xilian Wang, Liyuan Yang, Shaozhen Zhao, Hong Nian, Ruihua Wei

**Affiliations:** 1 Tianjin Medical University Eye Hospital, Eye Institute & School of Optometry and Ophthalmology, Tianjin, China; 2 Doheny Eye Institute, and Department of Ophthalmology, David Geffen School of Medicine, University of California Los Angeles (UCLA), Los Angeles, California, United States; 3 Tianjin Beichen Hospital, Tianjin, China

**Keywords:** adipose-derived mesenchymal stem cells (ADSCs), autoimmune dacryoadenitis, cytokines, Th17 responses, Th1/Th17 cells

## Abstract

**Purpose:**

To investigate the immunoregulatory roles of adipose-derived mesenchymal stem cells (ADSCs) in autoimmune dacryoadenitis.

**Methods:**

Rabbits were treated with ADSCs or phosphate-buffered solution on days 1, 3, 5, 7, and 9 after injection of activated peripheral blood lymphocytes, and clinical scores were determined by assessing tear production, break-up time, and fluorescein and hematoxylin and eosin staining. Inflammatory response was determined by measuring the expression of different mediators of inflammation in the lacrimal glands. The Th1/Th17-mediated autoreactive responses were evaluated by determining the proliferative response and the expression of cytokine genes and the lineage-determining transcription factors. The frequency of regulatory T cells (Tregs) was also examined.

**Results:**

The ADSC-treated rabbits showed decreased autoimmune responses, and the secretory function of their lacrimal gland was restored significantly. Treatment with ADSCs downregulated the Th1 and Th17 responses but enhanced Tregs function. In addition, ADSC treatment noticeably suppressed the expression of matrix metalloproteinase (MMP)-9, MPP-2, IL-1β, and IL-6, whereas it enhanced the expression of the anti-inflammatory cytokine IL-10.

**Conclusions:**

Our results demonstrated that ADSC administration efficiently ameliorates autoimmune dacryoadenitis mainly via modulating Th1/Th17 responses.

Primary Sjogren's syndrome (SS) is a chronic autoimmune disease characterized by lymphocytic infiltration of exocrine glands, which leads to functional impairment of the salivary and lacrimal glands.^1^ Whereas the progression of SS has typically been associated with Th1 cell phenotype, new studies have shown that altered Th17 responses also contribute to disease pathogenesis.^2,3^ In addition, aberrant T regulatory cells (Tregs) have also been reported in the salivary gland and peripheral blood of SS patients.^4^ Treatment for SS is mainly palliative relief, and many treatments are associated with adverse effects that limit their long-term utility.^5^ Hence, developing new therapies for SS is very much needed.

Mesenchymal stem cells (MSCs) have emerged as a potential strategy for treating autoimmune diseases, owing to their strong immunosuppressive activity.^6–10^ Xu et al.^11^ have recently reported the treatment effect of MSCs on SS in NOD mice, using allogeneic mesenchymal stem cells derived from bone marrows. Although bone marrow remains the main source for MSCs, adipose-derived MSCs (ADSCs), which can be easily isolated from fat tissues and effectively expanded, have become an attractive source of MSCs.^12^ Adipose-derived MSCs have been used in the treatment of some autoimmune diseases, such as collagen-induced arthritis, experimental colitis, and experimental autoimmune diabetes.^13–15^ In this study, we determined the effect of ADSCs on autoimmune dacryoadenitis,^16,17^ a rabbit model for human SS, which closely mimics human SS and has been exploited in evaluating new experimental therapies for SS.^18^

In this study, we addressed the therapeutic effect of ADSCs on autoimmune dacryoadenitis in rabbits and explored the underlying mechanisms. Our results demonstrated that ADSC administration efficiently ameliorates autoimmune dacryoadenitis mainly via modulating Th1/Th17 responses.

## Materials and Methods

### Animals

Adult New Zealand white rabbits (3.5–4 kg), female, were purchased from Vital River (Beijing, China). All rabbits were housed under pathogen-free conditions and maintained in a 12-hour light–dark cycle (8 AM–8 PM) in a temperature-controlled room (25°C ± 2°C) at 50% to 75% relative humidity without exposure to forced air for 1 week before experimental manipulation. All the animals were used in accordance with the ARVO Statement for the Use of Animals in Ophthalmic and Vision Research. All eyes were carefully examined before experimentation.

### Induction of Autoimmune Dacryoadenitis

The left inferior lacrimal gland (LG) was surgically excised under anesthesia for the isolation of purified LG epithelial cells (pLGECs) by using the methods described by Guo et al.^19^ In brief, peripheral blood was collected from rabbits for isolation of lymphocytes. The pLGECs and the peripheral blood lymphocytes (PBLs) were cultured separately for 2 days. Then, the pLGECs were irradiated and cocultured with equal numbers of autologous PBLs. Five days later, the activated PBLs were harvested and were adoptively transferred (1.5 × 10^6^ suspended in 100 μL sterile phosphate-buffered saline [PBS]) via ear margin vein into rabbits to induce autoimmune dacryoadenitis.

### Adipose-Derived MSC Isolation and Culture

Adipose tissues were obtained aseptically from rabbits under anesthesia, washed twice, minced, and incubated with 0.1% collagenase type I (Gibco, Grand Island, NY, USA) in PBS for 1 hour at 37°. After the termination of digestion, the samples were centrifuged. Afterwards the supernatant was discarded and the cell pellets were resuspended with culture medium (Dulbecco's modified Eagle's medium/F12 [1:1]) (Gibco) and 10% fetal bovine serum and incubated at 37°, 5% CO_2_. Later, the medium was replaced every 2 to 3 days; when the monolayer of adherent cells reached 80% confluence, cells were trypsinized and subcultured. For the experiments, we used the third to fourth passage of ADSCs.

### Adipose-Derived MSC Characterization by RT-PCR

Total RNA was extracted from 1 × 10^6^ cells of ADSC primary cultures (P1–P3) by using the Trizol reagent (Invitrogen, Carlsbad, CA, USA) according to manufacturer's instructions. The first-strand cDNA was synthesized with a reverse transcription kit (Fermentas, Burlington, ON, Canada). Gene-specific primers for CD29, CD34, CD44, CD45, CD73, and CD90 are listed in Table 1. Glyceraldehyde 3-phosphate dehydrogenase (GAPDH) was used as endogenous control.

**Table 1:**
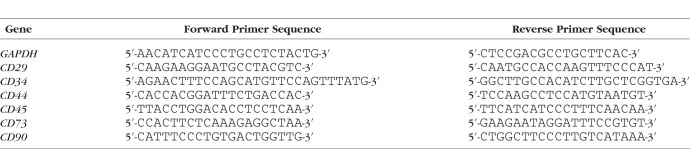
The Sequences of Primers Used in This Study for RT-PCR

### Adipose-Derived MSC Differentiation Assay

#### Adipogenic Induction.

Adipose-derived MSCs were seeded in 1 × 10^5^/mL in six-well plates with 2 mL medium, cultured, and changed to adipogenic differentiation medium (Cyagen, Suzhou, China). Three days later, the medium was replaced with maintenance medium for 24 hours, then changed back to induced medium. After three to five cycles (induced/maintenance medium), the cells were in maintenance medium for another 7 days. Then the cells were fixed and stained with Oil Red O. Adipogenesis was observed by the formation of lipid droplets following Oil Red staining.

#### Osteogenic Induction.

Adipose-derived MSCs were seeded in 1.5 × 10^4^/mL in six-well plates with 2 mL medium and changed to osteogenic differentiation medium (Cyagen) when the cells were approximately 60% to 70% confluent. The medium was changed every 3 days for 3 weeks. Then the cells were fixed and stained with alizarin red S. Osteogenesis was evident by the formation of alizarin-red–positive calcified deposits.

### Clinical Assessment of Autoimmune Dacryoadenitis

All rabbits were examined every 2 weeks with a slit-lamp biomicroscope for clinical signs of autoimmune dacryoadenitis after the first ADSC administration. Tear production was assessed by Schirmer's test with anesthesia. A Schirmer strip was inserted in the lower fornix of the eye for 1 minute and the length of the wetted area of the strip was measured. Tear break-up time (BUT) was measured to evaluate the tear film stability with a slit-lamp biomicroscope equipped with a blue filter.^18,20^ Five microliters of 2% fluorescein was applied in the middle of the lower eyelid and after several blinks the BUT was recorded in seconds. The cornea was divided into four quadrants and a standardized grading system was used to score the fluorescein staining separately. Scores from four regions were summed to a final grade (total, 16 points). Fluorescein staining was graded as follows^21^: absent, 0; slightly punctuate staining < 30 spots, 1; punctuate staining > 30 spots, but not diffuse, 2; severe diffuse staining but no positive plaque, 3; and positive fluorescein plaque, 4.

### Histologic Analysis of Lacrimal Glands

Rabbits were killed at the end of 6 weeks and OD inferior LGs were removed at necropsy. One part was fixed in 10% formalin and embedded in paraffin. The tissue was serially sectioned at 5-μm setting and six deeper sections were obtained approximately at 30-, 130-, 230-, 330-, 430-, and 530-μm depths. Depths were calculated from the surface of the tissue specimen. The sections obtained were taken for standard staining with hematoxylin and eosin (H&E), and then scanned and measured from the photographs, using CellSen software (Olympus, Tokyo, Japan). A focus was defined as an aggregate of >50 lymphocytes.^22^ The number of focus/4 mm^2^ of lacrimal tissue^23–25^ was calculated by a blinded pathologist with a BX51 microscope (Olympus).

### Treatment Protocols

To study the therapeutic effect of ADSC administration, rabbits were injected intravenously with 1 × 10^7^ ADSCs diluted in PBS (ADSC-treated group, *n* = 6) or with an equal volume of PBS (untreated group, *n* = 6). Normal rabbits were used as controls (control group, *n* = 6). The first injection of ADSCs or PBS was performed at 12 hours after adoptive transfer of activated PBLs (day 1), the next injections were performed every other day (day 3, 5, 7, and 9). This ADSC therapeutic protocol was based on our preliminary experiments, which have been found to be most effective for autoimmune dacryoadenitis in rabbits.

### Real-Time Quantitative PCR (qPCR)

Total RNA from cells or tissues was extracted by using the Trizol reagent (Invitrogen). The first-strand cDNA was synthesized with a reverse transcription kit (Fermentas). All gene transcripts were analyzed by qPCR with SYBR Green Master Mix (ABI; Applied Biosystems, Foster City, CA, USA) using an ABI 7900 HT Sequence Detection System. Gene-specific primers for real-time PCR are listed in Table 2. Glyceraldehyde 3-phosphate dehydrogenase was used as endogenous control in all experiments. For each sample, the relative abundance of target mRNA was calculated from the obtained Δ*Ct* values for both target and endogenous reference gene *GAPDH* by applying the following formula: relative mRNA expression = 2 ^[Δ^*^Ct^*^(control)–Δ^*^Ct^*^(target)]^.

**Table 2:**
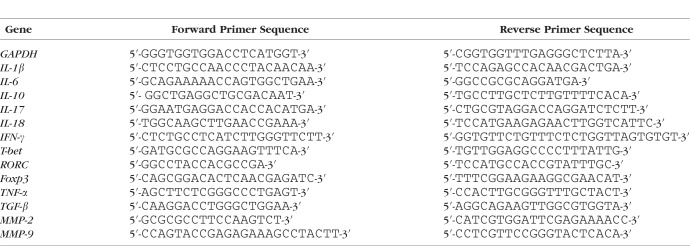
The Sequences of Primers Used in This Study for qPCR

### Flow Cytometric Analysis

The lymphocytes in LGs and spleens were fixed and permeabilized with Foxp3 staining buffer set (ebioscience, San Diego, CA, USA) according to the manufacturer's instructions. Then cells were stained with phycoerythrin (P)-labeled mAb anti-human Foxp3 (Biolegend, San Diego, CA, USA) and FITC-labeled mAb anti-rabbit CD4 (AbD, Raleigh, NC, USA) and then analyzed with FACScalibur flow cytometer (BD Biosciences, San Diego, CA, USA).

The splenic lymphocytes were labeled with 5 μM carboxyfluorescein diacetate succinimidyl ester (CFSE) and cocultured with ADSCs at different ratios in the presence of irradiated pLGECs for 5 days. Then the T-cell proliferation was measured by FACS analysis.

### Isolation of Lymphocytes From Rabbit LGs

Lacrimal glands were removed aseptically from rabbits under anesthesia, washed, minced and incubated with 0.1% collagenase type I (Gibco) in PBS for 30 minutes at 37° C. After incubation, the digested tissues were passed through 100-μm then 70-μm cell strainer and spun down at 108*g* for 5 minutes. The pellet was resuspended with 10 mL 40% Percoll. The cell suspension was gently overlaid onto 70% Percoll and centrifuged for 22 minutes at 800*g* (no brake). The lymphocytes were collected from the interface layer and washed by centrifugation in PBS. The pellet was resuspended in PBS and spun down at 500*g* for 5 minutes at 4°C. This process was repeated two times.^26^ The final pellet was resuspended in complete RPMI 1640 medium and kept on ice.

### Bromodeoxyuridine (BrdU) Assay

The PBLs (3 × 10^5^) in a total volume of 200 μL were cocultured with irradiated pLGECs (3 × 10^5^, 1.5 × 10^5^, 0.75 × 10^5^) at 37°C for 72 hours in 96-well tissue culture plates. For every experimental condition, each culture was performed in triplicate. T-cell proliferation was studied thereafter by measurement of BrdU incorporation, using a cell proliferation kit (Roche Diagnostics GmbH, Mannheim, Germany) according to the manufacturer's instructions.

### Western Blot Analysis

Frozen tissues were homogenized in ice-cold lysis buffer. The protein concentration was determined by using Bicinchoninic Acid (BCA) Protein Assay Kit (Biorega, Tianjin, China) following the manufacturer's procedure. Equal amounts of protein (100 μg) were loaded and separated by SDS-PAGE gel, and then transferred onto polyvinylidene difluoride membrane. Membranes were blocked in 5% fat-free milk, and then incubated with anti-Foxp3 primary antibody (1:100; Biolegend) that was diluted in 5% fat-free milk. Anti–β-actin (1:500; Biolegend) was chosen as a standard. After three washes with PBS-T, membranes were incubated with appropriate horseradish peroxidase-conjugated antibody (ZSGB-BIO, Beijing, China), then washed again and developed with ECL Prime Western Blotting Detection Reagent (GE, Little Chalfont, Buckinghamshire, UK). The bands were scanned by Multispectral Imaging System (UVP, Upland, CA, USA) and analyzed by Quantity One software (Bio-Rad, Hercules, CA, USA).

### Statistical Analysis

We used Graph Pad Prism 5.0 (GraphPad, San Diego, CA, USA) for statistical analyses. Data were presented as means ± SD. The *t*-test was used to compare the difference between groups. All tests were 2-tailed. Statistical significance was set at *P* < 0.05.

## Results

### Injection of Activated PBLs Induced Autoimmune Dacryoadenitis With Severe Lymphocytic Infiltration

After adoptive transfer of activated PBLs, the autoimmune dacryoadenitis in rabbits developed with regularity. The clinical symptoms usually appeared at 3 days after adoptive transfer, including reduced tear production, shortened tear BUT, and stained corneal epithelium with fluorescein. The disease became more evident at 2 weeks after transfer and was characterized by significantly decreased tear production and BUT, with significantly increased scores of fluorescein staining. The tear production and BUT remained almost unchanged thereafter. Scores of fluorescein staining peaked at week 6 and remained relatively constant thereafter. This chronic dry eye disease can persist 6 months after transfer of PBLs with evident ocular surface defects and severe lymphocytic infiltration.

### Isolation and Characterization of Rabbit ADSCs

**Figure 1:**
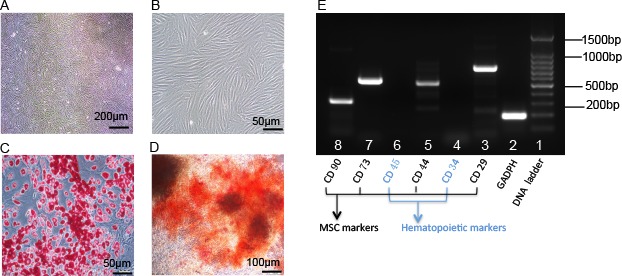
Characterization of ADSCs. (A, B) Morphology of ADSCs at passage 3. Most cells remained fibroblastic (spindle shaped) ADSCs upon subculturing to P3. (C) Adipogenic differentiation assay. Intracellular accumulated lipid droplets (red color in the image) in adipogenic ADSCs as revealed by Oil Red O staining. (D) Osteogenic differentiation assay. Vast extracellular calcium deposits (orange-red in the image) in osteogenic ADSCs as revealed by alizarin red S staining. (E) Reverse transcription–PCR analysis for MSC surface markers and hematopoietic markers. Total RNA was isolated from ADSCs (P3) and was subjected to RT-PCR. It showed that ADSCs were positive for the MSC markers CD29, CD44, CD73, and CD90, and negative for hematopoietic markers CD34 and CD45.

To evaluate whether ADSCs modulated autoimmune dacryoadenitis, we isolated and characterized ADSCs, as previous described.^27,28^ Adipose-derived MSCs at passage 3 (P3) showed a similar fibroblast-like morphology (Figs. 1A, 1B). Reverse transcription–PCR analysis shown in Figure 1E demonstrated that ADSCs were positive for the MSC markers CD29, CD44, CD73, and CD90, and negative for hematopoietic markers CD34 and CD45. The differentiation ability of ADSCs was determined by Oil Red O and alizarin red S staining. As shown in Figures 1C and 1D, ADSCs were able to differentiate into adipocytes and osteocytes.

### Adipose-Derived MSC Administration Alleviated Autoimmune Dacryoadenitis Clinically and Histologically

To investigate whether ADSCs have therapeutic effects on induced autoimmune dacryoadenitis, ADSCs or PBS was injected intravenously into rabbits on days 1, 3, 5, 7, and 9 after adoptive transfer of activated PBLs. The severity of disease was evaluated by assessing tear production, BUT, and fluorescein and H&E staining.

**Figure 2:**
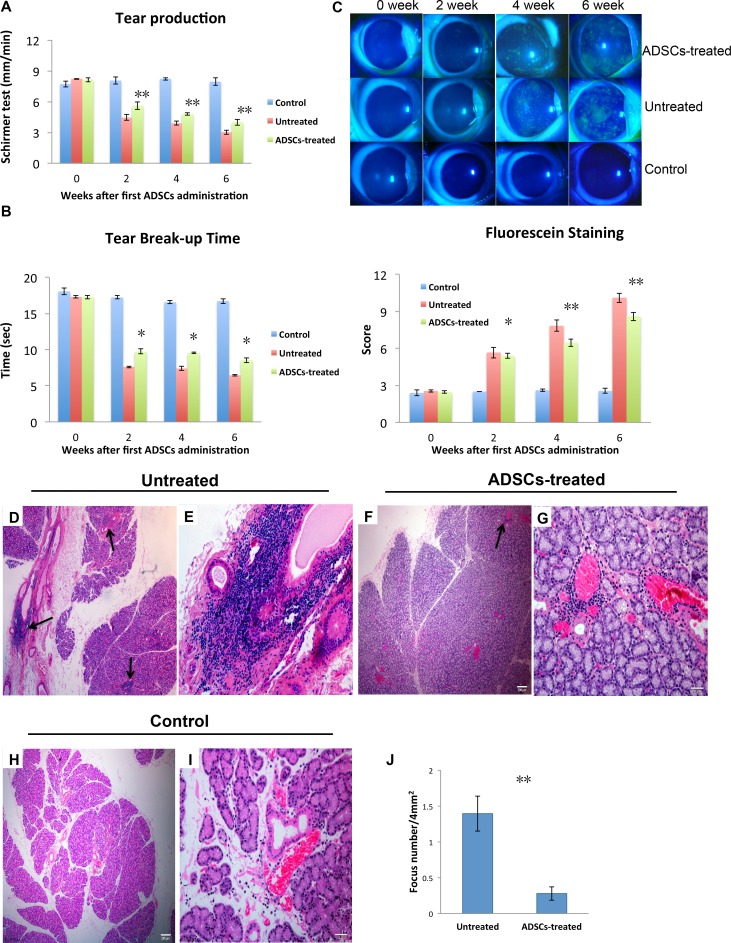
Adipose-derived MSCs alleviated clinical symptoms of autoimmune dacryoadenitis. Rabbits with autoimmune dacryoadenitis were injected intravenously with PBS (untreated group) or with 1 × 10^7^ rabbit ADSCs (ADSC-treated group) every other day starting on day 1 after transfer of activated PBLs. (A) Tear production. Schirmer test was performed at 0, 2, 4, and 6 weeks after first ADSC administration. (B) Tear break-up time demonstrates tear instability. Slit-lamp examination was performed in control, untreated, and ADSC-treated group. (C) Images and scores of fluorescein staining. Detection of ocular surface defects due to deficiency in preocular tear film protection was evaluated with fluorescein staining (top), and the intensity of staining of the cornea was scored (bottom). (D–J) Histologic analysis of lacrimal glands in rabbits. (D–I) The representative photographs (H&E staining) of lacrimal glands in untreated, ADSC-treated, and control rabbits are shown. (D, E) Substantial lymphocytic infiltration in the lacrimal glands of untreated group. Arrows indicate lymphocytic foci in the typical periductal and perivascular distribution (>2 foci per 4 mm^2^ of this representative LG section. The area (∼6 mm^2^) of the section (×40) examined was measured from the photographs, by using CellSen software. (F, G) Reduced lymphocyte infiltration in the lacrimal glands of the ADSC-treated group (<1 focus per 4 mm^2^ of this representative LG section). The area (∼9 mm^2^) of the section (×40) examined was measured from the photographs, by using CellSen software. (H, I) Occasional small lymphocytic aggregates in normal control group. (J) The numbers of lymphocytic foci in lacrimal glands were evaluated as described in Materials and Methods. The number of foci per 4 mm^2^ is shown. Data are presented as mean ± SD (A, B, C, J), n = 3. *P < 0.05, **P < 0.01. Data are representative of three independent experiments (A–J), six rabbits per group in each experiment.

As shown in Figure 2A, the mean aqueous tear volume of the ADSC-treated group was significantly higher than that of the untreated group at week 2, 4, and 6 after first ADSC administration (*P* = 0.00002, *P* = 0.0002, and *P* = 0.00001, respectively).

As summarized in Figure 2B, BUT scores in the ADSC-treated group were significantly higher than in the untreated group at week 2, 4, and 6 after first ADSC administration (*P* = 0.0006, *P* = 0.0002, and *P* = 0.0005, respectively).

Fluorescein staining scores increased 2.2-fold 2 weeks after first ADSC administration, with no significant difference between the untreated and ADSC-treated group (Fig. 2B). The animals' condition appeared to be improved at week 4 and 6. Although scores in the ADSC-treated group remained 2.5- and 3.4-fold greater than the control value at week 4 and 6, they were 17% and 15% lower than in the untreated group (*P* = 0.0004 and *P* = 0.002, respectively).

Examination of H&E-stained sections (Figs. 2D–I) revealed that the inferior LGs from the ADSC-treated group had only rare immune cell infiltrates, whereas those from the untreated group were heavily infiltrated and the immune cells were frequently concentrated around the ducts and venules.

### Adipose-Derived MSC Treatment Inhibited the Proliferation of Autoreactive T Cells In Vitro and In Vivo

**Figure 3:**
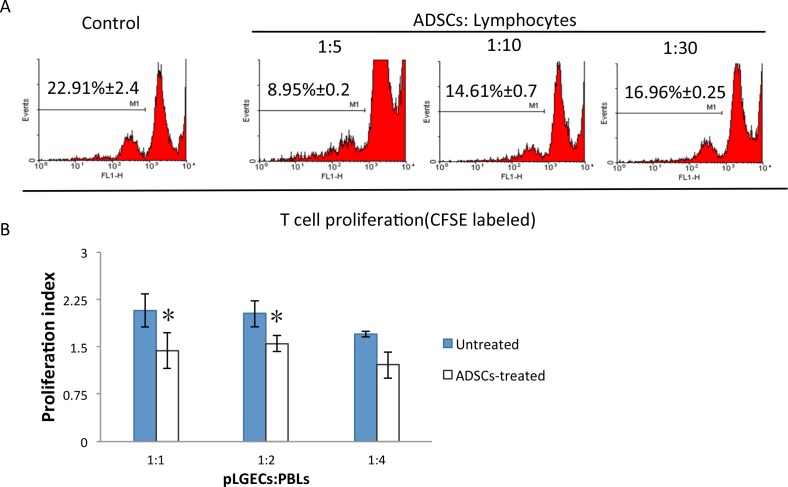
Adipose-derived MSC treatment inhibited the proliferation of pathogenic T cells in vitro and in vivo. (A) Adipose-derived MSCs' effect on T-cell proliferation. Splenic lymphocytes labeled with 5 μM CFSE were cultured with irradiated pLGECs in the presence of ADSCs at different MSC: T-cell ratios of 1:5, 1:10, and 1:30. After 5 days, the lymphocytes were harvested and subjected to FACS analysis. (B) The proliferation index of PBLs from ADSC-treated or untreated rabbits was determined by BrdU assay. Data are representative of three independent experiments, and bar graphs show mean ± SD. *P < 0.05, **P < 0.01.

To determine the mechanism by which ADSC administration ameliorated the induced LG inflammation and tissue damage, we investigated the effect of ADSCs on activation of autoreactive T cells. The splenic lymphocytes of disease-induced rabbits were isolated and prelabeled with 5 μM CFSE. Then they were stimulated in vitro with irradiated pLGECs for 5 days, in the absence or presence of ADSCs. The activated T cells were separated with ficoll by density gradient centrifugation before subjecting them to FACS analysis. As shown in Figure 3A, the T cells, cultured in the presence of ADSCs, showed significantly reduced rates of proliferation in a dose-dependent manner. To determine whether ADSCs also have a suppressive effect on PBLs in vivo, the PBLs were isolated from treated or untreated rabbits and subjected to BrdU proliferation assay. As shown in Figure 3B, the proliferation rates of PBLs from the ADSC-treated group were significantly lower than those from the untreated group.

### Adipose-Derived MSC Treatment Suppressed the Expression of Inflammatory Mediators in the LGs of Dacryoadenitis Rabbits

**Figure 4:**
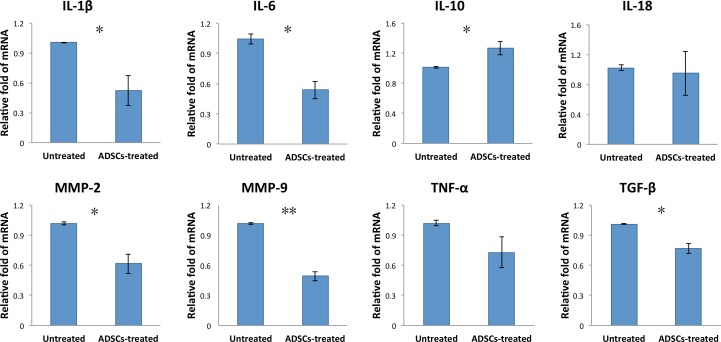
Adipose-derived MSC treatment regulated the expression of pro- and anti-inflammatory cytokines and chemokines. Lacrimal glands were obtained from untreated and ADSC-treated rabbits at 6 weeks after first ADSC administration, and IL-1β, IL-6, IL-10, IL-18, TNF-a, TGF-β, MMP-2, and MMP-9 mRNA expression was measured by qPCR. Data are representative of three independent experiments and bar graphs show mean ± SD, n = 3. *P < 0.05, **P < 0.01.

We next investigated the effect of ADSC treatment on expression of inflammation mediators that are linked to the inflammation of SS. The LGs were collected from untreated or ADSC-treated rabbits and subjected to qPCR assay for the expression of inflammation-related molecules. As indicated in Figure 4, ADSC injection significantly reduced the expression of inflammatory mediators, including IL-6, IL-1β, TGF-β, matrix metalloproteinase (MMP)-2, and MMP-9, while it increased the expression of the anti-inflammatory cytokine IL-10 in the LGs of autoimmune dacryoadenitis rabbits. However, ADSC treatment had no effect on the expression of IL-18 and TNF-a mRNA.

### Adipose-Derived MSC Treatment Downregulated Th1 and Th17 Autoimmune Responses

**Figure 5:**
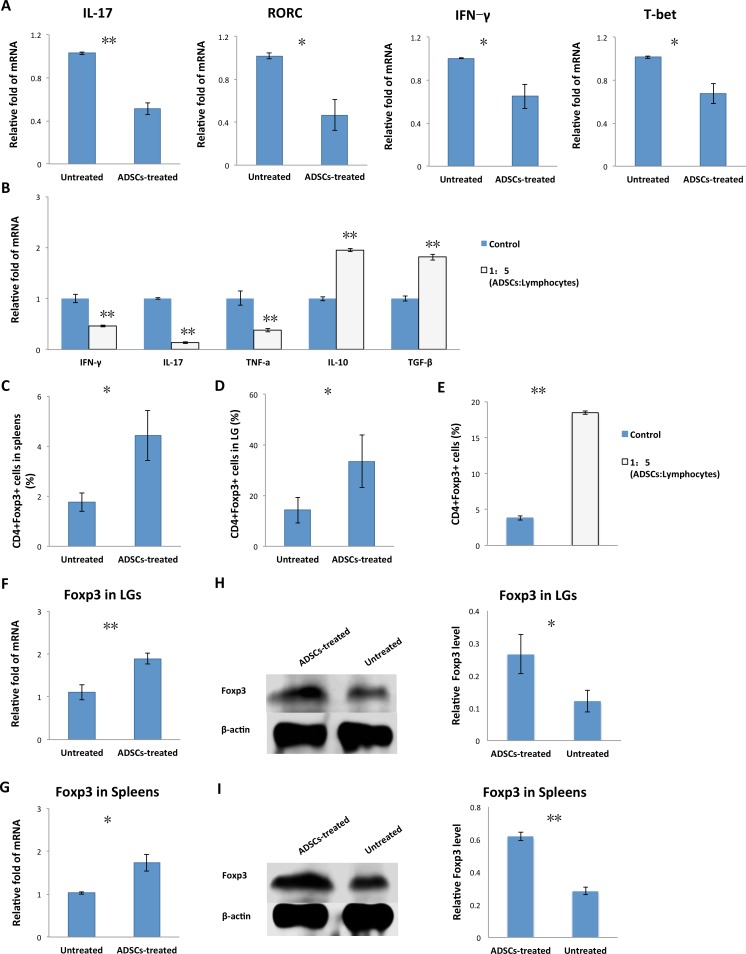
Adipose-derived MSC treatment regulated Th1/Th17/Tregs in vivo and in vitro. (A) Lacrimal glands were removed from untreated and ADSC-treated rabbits, and IL-17, IFN-γ, T-bet, and RORC mRNA expression was analyzed by real-time qPCR. (B) Adipose-derived MSCs cocultured with splenic lymphocytes at the ratio of 1:5 were collected. The mRNA expression levels of IL-10, IFN-γ, IL-17, TNF-α, and TGF-β were measured by qPCR. (C) Percentage of CD4^+^Foxp3+ cells among splenic lymphocytes isolated from ADSC-treated and untreated rabbit spleen. (D) Percentage of CD4^+^Foxp3+ cells among lymphocytes isolated from LGs of ADSC-treated and untreated rabbits. (E) Adipose-derived MSCs cocultured with splenic lymphocytes at the ratio of 1:5 were measured by flow cytometry for the percentages of CD4^+^Foxp3+ cells at day 5. (F, G) Quantitative PCR analysis of Foxp3 mRNA expression in LGs and spleens of ADSC-treated and untreated rabbits. (H, I) Immunoblot of LGs and spleens of ADSC-treated and untreated rabbits. Tissue lysates were immunoblotted with indicated antibodies. Left: Levels of Foxp3 and β-actin. Right: Relative Foxp3 level = Foxp3 level/β-actin level. Data are representative of three independent experiments and bar graphs show mean ± SD, n = 3. *P < 0.05, **P < 0.01.

Given that both Th1 and Th17^29^ autoimmune responses contribute to the pathogenesis of SS, we examined whether ADSC treatment affected Th1/Th17 cell responses. The LGs from untreated or ADSC-treated rabbits were analyzed for expression of Th1/17 cytokine genes (IFN-γ, IL-17) and the lineage-determining transcription factors (T-bet, RORC). As shown in Figure 5A, the ADSC-treated group had significantly lower mRNA expression of IL-17 (0.51 ± 0.06 versus 1.03 ± 0.01, *P* = 0.006), RORC (0.47 ± 0.15 versus 1.02 ± 0.03, *P* = 0.029), T-bet (0.67 ± 0.09 versus 1.01 ± 0.01, *P* = 0.027), and IFN-γ (0.64 ± 0.21 versus 1.01 ± 0.01, *P* = 0.027) than the untreated group, suggesting that ADSCs suppressed both Th1 and Th17 responses concurrently in vivo.

We also examined the effect of ADSCs on the function of Th cells and expression of the inflammatory cytokines in vitro. Splenic lymphocytes isolated from disease-induced rabbits were cocultured with irradiated pLGECs in the presence or absence of ADSCs. Then the mRNA levels of IL-10, IFN-γ, IL-17, TNF-α, and TGF-β in the splenic lymphocytes were assessed by using qPCR. As shown in Figure 5B, ADSC administration significantly inhibited the expression of IFN-γ (0.47 ± 0.02 versus 1.00 ± 0.09, *P* = 0.005), IL-17 (0.14 ± 0.02 versus 1.00 ± 0.01, *P* = 0.001), and TNF-α (0.38 ± 0.034 versus 1.01 ± 0.14, *P* = 0.002), but significantly enhanced the expression of anti-inflammatory cytokines IL-10 (1.95 ± 0.03 versus 1.00 ± 0.04, *P* = 0.001) and TGF-β (1.81 ± 0.06 versus 1.00 ± 0.05, *P* = 0.001).

### Adipose-Derived MSC Treatment Promoted the Proportion of Tregs In Vivo and In Vitro

To evaluate whether ADSC treatment affects Tregs function, we analyzed the frequency of CD4^+^Foxp3+ T cells in the spleens and LGs from disease-induced rabbits, with or without ADSC treatment. As shown in Figures 5C and 5D, the percentage of CD4^+^Foxp3+T cells was greatly higher in the spleens and LGs of ADSC-treated rabbits than in those of the untreated rabbits (4.43% ± 1.01% versus 1.77% ± 0.38%, 33.58% ± 10.43% versus 14.26% ± 5.05%, respectively). Further qPCR analysis revealed that Foxp3 mRNA expression was significantly elevated in the spleens and LGs of ADSC-treated rabbits compared with the untreated rabbits (1.72 ± 0.19 versus 1.03 ± 0.03, 1.89 ± 0.13 versus 1.11 ± 0.18, respectively; Figs 5F, 5G). Western blot analysis showed that there was a significant increase in Foxp3 protein expression in spleens and LGs of ADSC-treated rabbits compared with the untreated rabbits (Figs. 5H, 5I).

We also examined the effect of ADSCs on Tregs in vitro. Splenic lymphocytes isolated from induced dacryoadenitis rabbits were cocultured with irradiated pLGECs in the presence or absence of ADSCs. After 5 days, the cells were harvested and subjected to FACS assay. As indicated in Figure 5E, the percentages of CD4^+^Foxp3+ T cells were significantly higher in the ADSC-treated group than in the control group (18.47% ± 0.25% versus 3.83% ± 0.31%, respectively).

## Discussion

Mesenchymal stem cell transplantation has been recognized as one of the potential strategies to treat autoimmune disorders.^30,31^ However, the acquisition of MSCs from bone marrow (BMSCs) is a painful procedure, and the ability of differentiation of BMSCs decreases with age.^32^ As compared with BMSCs, ADSCs are more easily accessible. Recent studies^33,34^ have shown that ADSCs are as effective suppressors of immune response as BMSCs. Previous studies have reported successful treatment of SS disease in the NOD mice by using allogeneic BMSCs; however, the therapeutic mechanisms by which ADSCs suppress induced autoimmune dacryoadenitis remain unclear. In this study, we demonstrated that allogeneic ADSCs are effective for treating autoimmune dacryoadenitis in rabbits.

In other experimental models of autoimmune diseases, such as colitis and autoimmune diabetes, ADSC treatment has been shown to suppress CD4^+^ immune responses.^13,35^ The results shown here are consistent with these studies. We found that administration of ADSCs to rabbits with induced autoimmune dacryoadenitis resulted in a decreased T-cell–mediated response. Treatment with ADSCs downregulated T-cell proliferative responses and the expression of Th1 and Th17 cytokine genes in vivo and in vitro. Our results here are in accordance with a recent study showing that the injection of murine BMSCs into mice with SS prevented disease progression by suppressing Th17 cells.^11^ We additionally showed that ADSCs also suppressed Th1 response. Given that both Th1 and Th17 autoimmune responses contributed to the pathogenesis of SS disease, it is likely that ADSCs might be a better candidate for therapy of human SS.

Matrix metalloproteinases are a family of proteolytic enzymes that are engaged in pathologies associated with infections, tumors, and autoimmune disorders.^36^ Previous studies^37–39^ have demonstrated that MMPs are involved in the pathogenesis of SS. Lozito et al.^40^ have demonstrated that BMSCs inhibit levels of exogenous MMP-2 and MMP-9 through TIMP-2 and TIMP-1, respectively. In the current study, we found that ADSC treatment significantly downregulated the mRNA expression of MMP-2 and MMP-9 in the LGs of dacryoadenitis rabbits. Decreased production of MMP-2 and MMP-9 could lead to reduced leukocyte recruitment to the LGs, resulting in diminished inflammatory infiltrates in the LGs of dacryoadenitis rabbits treated with ADSCs.

The development of Th1 and Th17 cells is principally related to the induction of transcriptional factors such as T-bet and RORγt, respectively.^41^ We determined the effect of ADSCs on the expression of these key factors associated with Th cell lineage differentiation and found that ADSC treatment significantly suppressed the expression of RORC and T-bet. These findings suggest that ADSC treatment may inhibit Th cell lineage commitments in vivo and thus ameliorate disease development, which is in agreement with the recent findings in the murine model by Mohammadzadeh et al.^42^

Th cell differentiation program is regulated by cytokines produced by innate immune cells. Interleukin 1β and IL-6 possess an enhancing effect on the differentiation and survival of Th17 cells.^43^ We observed that the mRNA expression of IL-1β and IL-6 was significantly lower in the LGs of the ADSC-treated group. The data suggest that ADSC treatment may form a cytokine milieu that suppresses the differentiation and function of Th17 cells. Indeed, we found that ADSCs possessed the ability to inhibit the expression of IL-17 in vitro.

CD4^+^CD25^+^Tregs play an important role in maintaining tolerance to self-antigens controlling occurrence of autoimmune diseases,^44,45^ and decreased frequency and reduced function of Tregs are associated with SS patients. Previous studies^46–48^ have demonstrated that ADSCs promote Tregs cells in vitro and in vivo. Consistent with these reports, we observed higher frequency of Tregs and expression of Foxp3 in the spleens and LGs of the ADSC-treated group than the untreated group, and found that ADSCs induced the expansion of Tregs in vitro. Furthermore, the expression of IL-10, a signature cytokine for Tregs that suppresses Th1/Th17 responses,^49,50^ was dramatically upregulated in the LGs of dacryoadenitis rabbits treated with ADSCs. Interleukin 10 plays a critical role in the control of antigen-reactive T cells, and the induction of peripheral tolerance in vivo.^51^ Interleukin 10 has also been found to be critical in the generation of Tregs.^52^ The observed increase in IL-10 may partially explain the increased induction of Tregs in the ADSC-treated group. Together, these data suggest that ADSCs could induce IL-10–producing Tregs in autoimmune dacryoadenitis rabbits, which may result in decreased function of Th1/Th17 cells.

In summary, our study demonstrated that systemic ADSC administration efficiently attenuated the clinical severity and diminished the inflammation of induced autoimmune dacryoadenitis in rabbits by suppressing Th1/Th17 responses and favoring Tregs expansion. This study has implication for further investigation of ADSCs in the treatment of related human disease.
